# Optimal bus bridging service for urban rail transit disruptions with stochastic passenger demand

**DOI:** 10.1371/journal.pone.0333686

**Published:** 2025-10-07

**Authors:** Yicheng Liu, Tao Yang, Juan Su

**Affiliations:** 1 Sichuan Provincial Engineering Technology Research Center for Modern Road Traffic Safety Assurance, Chengdu, China; 2 Chengdu Metro Operation Co., Ltd, Chengdu, China; 3 School of Transportation Engineering, Chang’an University, Xi’an, China; Southwest Jiaotong University, CHINA

## Abstract

Disruptions in urban rail transit (URT) systems can significantly impact operational efficiency, while well-designed bus bridging service (BBS) can effectively mitigate such effects. To address the surge in travel demand caused by disruptions, this study comprehensively considers alternative transportation modes that affected passengers may adopt (including taxis, shared bicycles, bridging buses, and walking), aiming to minimize both the operational costs of bridging buses and the total travel time of passengers. A travel choice model based on the random regret minimization (RRM) theory is developed to characterize passengers’ decision-making behavior following station disruptions. Demand uncertainty is represented using trapezoidal fuzzy variables, and a distributionally robust credibility optimization model is established. An innovative reinforcement learning-based parallel genetic algorithm (RPGA) is proposed for solving the model. A case study of a bidirectional disruption during the 08:00–10:00 on the section of Xi’an Metro Line 2 demonstrates that: (1) The proposed model exhibits stronger robustness under demand uncertainty, achieving a reduction of 3 dispatched vehicles and a cost saving of 9,439 RMB by moderately increasing passenger costs by 850 RMB and extending bridging time; (2) The RPGA algorithm outperforms Non-dominated Sorting Genetic Algorithm II (NSGA-II), Reinforcement Learning-based NSGA-II (RLNSGA-II), and Multi-objective Particle Swarm Optimization Algorithm (MOPSO) in hypervolume (HV), generational distance (GD), and non-dominated ratio (NDR); (3) Increasing the rated passenger capacity within a certain range can reduce average passenger delays but correspondingly raises transportation costs. This method effectively enhances the system’s ability to cope with demand fluctuations and provides decision-making support for emergency scheduling in urban rail transit.

## 1. Introduction

As urbanization accelerates, urban transportation systems are encountering unprecedented challenges. High-capacity and low-emission Urban Rail Transit (URT) has emerged as the backbone of urban public transportation networks. However, URT operations are frequently disrupted by equipment failures and other factors, particularly prolonged stoppages, which can result in mass passenger stranding and significantly reduce transportation efficiency. During such disruptions or emergencies, the capacity of rail systems often falls short of meeting passenger demand, leading not only to train delays but also to passengers being stranded on disrupted lines or at stations, further extending their travel times. For instance, statistics reveal that between 2018 and 2019, URT passengers in London experienced over 30 million hours of delays [1]. In 2022, mainland China’s urban rail transit system recorded more than 717 delays [2]. Similarly, Hong Kong’s MTR system faces an average of about 250 disruptions annually, with disruptions caused by power failures, signaling issues, and door malfunctions lasting over 60 minutes on average [3].

In the face of unplanned disruptions, URT operators typically adopt two strategies. Firstly, they make adjustments within the rail system by redirecting passengers to alternative URT lines for transfers to neighboring stations. Secondly, they invoke the bus system to evacuate stranded passengers. Research indicates that internal adjustments can effectively transfer passengers in short-term emergencies. However, when disruptions exceed 30 minutes, solely relying on internal adjustments becomes challenging to meet passenger demand. Consequently, activating bus bridging services becomes necessary [4]. According to Pender et al., 85% of agencies rely on bus bridging services to manage disruptions. Therefore, exploring innovative connections to ensure seamless and passenger-friendly transportation during unforeseen disruptions is crucial for achieving the city’s long-term sustainability goals [5].

A significant amount of academic attention has been devoted to the Bus Bridging Service Design Problem (BBSDP), which primarily aims to ensure the efficient and orderly evacuation of stranded passengers resulting from URT service disruptions. The BBSDP was first formulated by Kepaptsoglou and Karlaftis using a three-stage genetic algorithm approach for route generation, selection, and bus allocation [6]. Subsequent studies developed alternative optimization frameworks: Jin et al. proposed a column-generation and network-flow based method [7]. Wang et al. created a collaborative URT-bus dispatch model [8]. Gu et al. introduced a two-stage WSPT heuristic for time-delay tradeoffs [9], while Dou et al. minimized combined passenger/bus operation costs [10]. These models can be broadly categorized into deterministic and uncertain models, based on whether or not uncertainty is factored into the optimization process. Deterministic models rely on fixed parameters for planning bus schedules and predominantly adopt a passenger-centric perspective. This includes considerations such as passenger transfer and violation behaviors [11], spatial and temporal passenger demands [12], dynamic passenger flows during disruptions [13], and individual passenger path selection behaviors [14]. These studies collectively contribute a robust theoretical foundation and practical insights for addressing the BBSDP.

However, the effectiveness of the BBSDP in addressing disruptions is significantly impacted by various parameters, including minor fluctuations in bus operating times, which can render a deterministic BBSDP approach suboptimal or even impractical. Consequently, it is imperative to incorporate the impact of uncertainty. Uncertainty modeling represents an extension of deterministic modeling, focusing on the inclusion of uncertainties [15]. Recent studies have identified several uncertainties, such as disruption duration [16], passenger demand [17], and bus running time [18]. For instance, Wang et al. incorporated stochastic commuter demand and modeled it as a batch queuing process with hesitation and repetition characteristics using compound Poisson process theory [19]. Zhang and Lo formulated an optimization problem to determine the optimal initiation time for Shuttle Bus (SB) services under uncertain recovery duration, with the objective of minimizing expected total system costs [3]. Liang et al. developed a bus bridging optimization model addressing bus travel time uncertainty to minimize combined passenger and operational costs [18]. Xu et al. proposed a distributionally robust optimization model for rail transit tactical planning strategy design and disruption tolerance enhancement considering downtime uncertainty [20]. Luo et al. established a stochastic programming model accounting for uncertainties in both commuter demand and spare capacity of existing rail/bus lines, aiming to minimize unmet commuter demand [21]. Chen et al. introduced a robust optimization model based on Passenger Guidance and Extended Bus Bridging Service (E-BBS) to ensure reliable travel guidance and E-BBS solutions, specifically addressing the operational challenges posed by high uncertainty in bus running times [22].

The BBSDP is inherently NP-hard, posing significant challenges in solving large-scale instances. To enhance computational efficiency and identify near-optimal solutions, researchers have devised a range of meta-heuristic algorithms, including genetic algorithms [7], Tabu Search [23], and the Non-Dominated Sorting Genetic Algorithm II (NSGA-II) [9]. These algorithms excel at thoroughly exploring the solution space and maintaining search effectiveness, even when encountering potentially suboptimal or infeasible solutions. For a thorough grasp of research pertaining to bus bridging services in the context of URT disruption management, we recommend consulting the literature review [24]. Furthermore, for the reader’s convenience, representative research outcomes mentioned in this section have been neatly organized and presented in Table 1.

**Table 1:** Summary of BBSDP-related studies.

Author(s) (year)	Methodology	Objective function	Solution algorithm	Uncertainty
Wang (2019) [13]	Multi-objective optimization model	Minimize total passenger waiting time and minimum total number of buses dispatched	NSGA-II algorithm	Bus running time
Liang (2019) [18]	Robust optimization	Minimizing total ridership and operating costs	Column generation procedure	Bus running time
Xu et al. (2021) [20]	Distributionally robust optimization	Maximize the worst-case expected downtime	CPLEX	Disruption location, disruption duration
Luo and Xu (2021) [21]	Stochastic programming model	Minimize the expected unsatisfied demand	Sample average approximation	Commuter demand and spare capacities of existing rail and bus lines
Chen (2024) [22]	Robust models	Minimize passenger travel costs and operator costs	Dynamic decision framework	Bus running time
Wang et al. (2016) [8]	Integer linear programming	Minimize the total evacuation cost of the feeder-buses	Heuristic algorithm	N
Gu et al. (2018) [9]	Two-stage model	Minimize bus bridging time and reducing passenger delay	Heuristic algorithm	N
Dou et al. (2019) [10]	Mixed-integer nonlinear programming model	Minimize passenger inconvenience	A decomposition method	N
Zhang (2024) [11]	Integrated optimization model	Maximize bridging efficiency Minimize passenger waiting time	Two-stage genetic algorithm	N
Shao (2022) [12]	Optimization model	Minimize passenger waiting time	CPLEX	N
Zhu (2024) [14]	Mixed-integer nonlinear programming model	Minimize the sum of passenger travel time costs and fines associated with unserved passengers	Variable neighborhood search algorithm	N
Wang (2023) [25]	Integer linear programming	Minimize operator-oriented and passenger-oriented costs	Column generation method	N
Feng (2024) [26]	Mixed-integer nonlinear programming model	Minimize total passenger delay	Branch bounding algorithm	N
Our work	Multi-objective optimization model	Minimize the transportation cost of emergency bus vehicles and Minimize the average delay time of passengers	Reinforcement-learning-based Parallel Genetic Algorithm	Uncertain passenger demand

The extant scholarship on bus bridging services predominantly addresses scheduling paradigms within deterministic frameworks, with limited exploration into optimizing bridging modalities under stochastic demand conditions. Although advancements in multi-objective optimization algorithms have been achieved, their computational efficacy necessitates further refinement through integration with emerging technologies such as parallel computing. In response, this study makes three pivotal contributions:

(1) Development of a Multi-Objective Emergency Bus Scheduling Model: Formulated to concurrently minimize buses transportation costs and passenger travel costs, this model establishes a theoretical foundation for resolving real-world scheduling challenges.
(2) Innovative Quantification of Demand Uncertainty: By synthesizing interval-valued parameter possibility distributions with robust uncertainty sets, the original optimization problem is reformulated as a computationally tractable robust peer-to-peer model, effectively mitigating the complexities of demand stochasticity.
(3) Reinforcement-Learning-Based Parallel Genetic Algorithm (RPGA): A hybrid algorithm designed to efficiently solve the bi-objective model, RPGA introduces a novel methodological framework for emergency bus bridging optimization.

The paper is organized with Section 2 detailing the model formulation, including problem description and content of model construction; Section 3 presenting the designed algorithms for solving multi-objective models; Section 4 analyzing the model through a case study and conducting sensitivity analysis among the major factors; and Section 5 summarizing the conclusions of the paper and presenting the future outlook.

## 2. Mathematical formulation

### 2.1. Problem description

**Fig 1:**
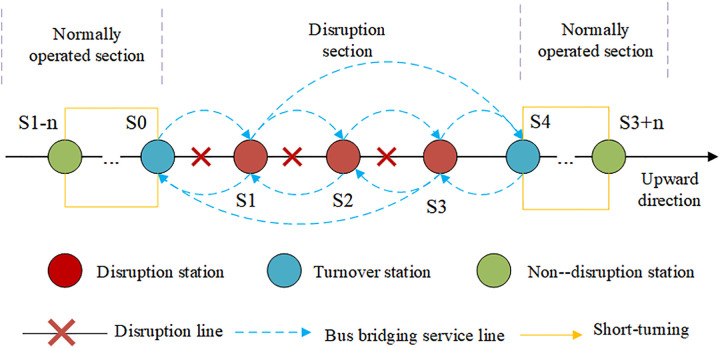
Schematic diagram of bus bridging service under single-line interruption in URT.

Fig 1 presents a schematic diagram of sudden service disruption on a single urban rail transit line, when a service disruption occurs between stations S1 and S3 in S1 File, the affected segment is defined as the disruption segment. The nearest turnback stations flanking this segment—S0 and S4—are dynamically designated for short-turning operations, with trains executing turnbacks at these stations and their adjacent counterparts. The bus bridging service is activated immediately after urban rail transit disruption and ends once the disruption is resolved. Building on the demonstrated advantages of new energy buses in environmental benefits (zero emissions, low noise), operational efficiency (low energy consumption, high performance), and sustainable development (reduced fossil fuel dependence), this study proposes a bus bridging service route system using new energy buses for adjacent disruption stations, which aligns with urban bus electrification development strategies and low-carbon transportation policy directives.

**Fig 2:**
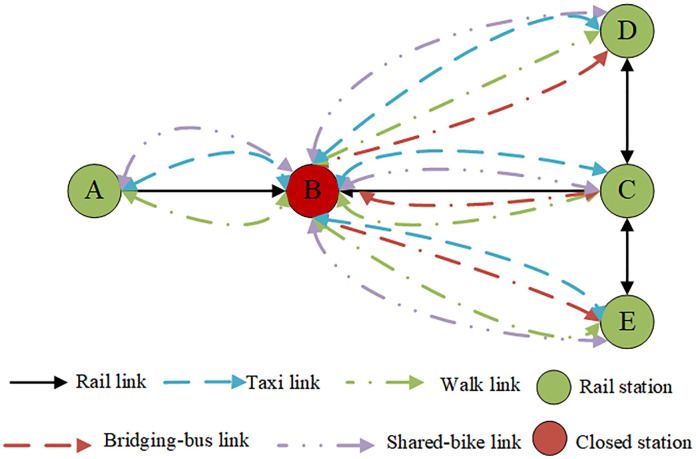
Mode choice under rail transit disruption.

As illustrated in Fig 2, the temporary closure of Station B has necessitated alternative travel arrangements for affected metro passengers. Commuters can no longer board or disembark at this station, requiring those who intended to depart from Station B to either select a neighboring station as their new starting point, await the station’s reopening, or utilize other transit options. Similarly, passengers traveling to Station B must now choose between alternative destination stations or different modes of public transportation. These decisions are informed by both personal travel experience and official guidance provided by transit authorities regarding available alternatives such as taxis, bike-sharing systems, shuttle services, or walking routes. The disruption has particularly impacted travel patterns, with all affected passengers needing to adjust their journeys based on these modified transportation options.

Metro operators must implement emergency response measures to maintain connectivity when stations are temporarily closed, typically by deploying shuttle bus services that integrate with alternative transport modes including new energy buses, taxis, bike-sharing systems, and pedestrian routes. This disruption scenario presents two key optimization challenges: (1) dynamic scheduling of new energy shuttle buses requiring optimal dispatch frequency and charging plans that minimize operational costs while accounting for variable energy consumption during traffic congestion, and (2) robust passenger flow distribution across available transport options considering uncertain demand patterns and travelers’ mode choice preferences based on time, cost and comfort factors. The problem is formulated as a dual-objective optimization model simultaneously minimizing bus transportation costs (energy consumption and fleet scheduling expenses) and passenger travel costs (waiting time and travel expenses), requiring solutions that maintain system resilience during peak demand fluctuations while ensuring sustainable operation of the temporary transport network.

### 2.2. Assumptions

The modeling framework is predicated on the following assumptions:

(1) Bus Stop Location Setting: Candidate bus stop locations are assumed to be set parallel to existing URT stations to facilitate quick transfers for passengers between the two traffic modes.
(2) Mode Choice: When selecting transfer modes (bus, taxi, bike-sharing, or walking), passengers comprehensively compare time, cost, and comfort rather than making random choices.
(3) Emergency Bus Type and Capacity: All emergency buses are assumed to be of the same type, with charging costs and maximum passenger capacity treated as fixed values, ignoring fluctuations in electricity prices across different time periods.
(4) Road Disruption Neglect: Potential disruptions to emergency bus operations—such as traffic accidents or road closures—are not considered.
(5) Emergency Bus Service Scope: Each emergency bus is restricted to serving only one specific origin-destination (OD) pair and is not allowed to stop at other stations to pick up passengers during its service period, ensuring operational efficiency and accuracy.

### 2.3. Symbol definitions

The relevant symbols in this paper are defined as shown in Table 2:

**Table 2:** Symbol description.

Symbol	Definition
S	Set of interrupted rail transit stations (s∈S)
B	Set of new energy buses (b∈B)
R	Set of emergency bus routes (r∈R)
A	Set of road network links (a∈A)
M	Set of travel modes (m∈M)
T	Set of time intervals (t∈T)
ψ	Unit electricity cost (RMB/kWh)
$c_{f}$	Fixed operating cost per unit time for buses (RMB/h)
τ	Time required for a single charging session (h)
α、β、γ	Passenger cost weighting coefficients (time, economic, comfort)
$E_{b}\left(v_{a}\right)$	Energy consumption function of vehicle b at speed $v_{a}$ (kWh/km)
$k_{1}$, $k_{2}$	Quadratic term coefficient and constant term of the energy consumption function
$\eta_{m}$	Motor efficiency (%)
$\eta_{c}$	Charging efficiency (%)
$Q_{b}^{\max}$	Maximum battery capacity of vehicle b (kWh)
$v_{a}^{free}$	Free-flow speed of link a (km/h)
$C_{a}$	Capacity of link a (veh/h)
$L_{a}$	Length of link a (km)
$D_{a}$	Total travel distance of route r (km)
${\tilde{d}}_{s}$	Fuzzy demand interval for station s (${\tilde{d}}_{s}\in \left[{\underset{―}{d}}_{s},{\overset{―}{d}}_{s}\right]$)
θ	Credibility level threshold (θ∈[0,1])
μ	Regret sensitivity parameter in the RRM model
$U_{s,m}$	Random utility function for mode m at station s
$R_{s,m}$	Regret value for mode m
$g\left(l_{s,m}\right)$	Comfort penalty function for walking distance l
$v_{a}\left(x_{a}\right)$	Speed-flow function for link a
$f\left(x_{a}\right)$	Generalized link impedance function
Cr(A)	Credibility measure of event A
$\pi \left({\tilde{d}}_{s}\right)$	Possibility distribution of fuzzy demand ${\tilde{d}}_{s}$
$t_{s,m}$	Total travel time for mode m from station s to destination
$c_{s,m}$	Economic cost for mode m (RMB)
$l_{s,m}$	Access walking distance for mode m (km)
λ	Weighting coefficients for multi-objective optimization (λ∈[0,1])
$y_{s,r}^{b}$	Departure frequency of new energy bus b on route r from station s (trips/h)
$x_{s,m}^{p}$	Passenger proportion choosing mode m at station s ($x_{s,m}^{p}\in \left[0,1\right]$)
$z_{b,t}$	Charging status of vehicle b during time interval t (1=charging, 0=not charging)
$\delta_{a,r}^{b}$	Whether vehicle b passes link a on route r (1=yes, 0=no)

### 2.4. Deterministic modeling

The objective function of this research comprises two components: the first part accounts for the transportation costs of bus bridging services between disrupted stations and other stations, including both energy consumption costs and temporal costs:


$$\min F_{1}=\sum_{b\in B}\left[\psi \cdot \sum_{a\in A}\frac{L_{a}}{v_{a}\left(x_{a}\right)}\;\cdot E_{b}\left(v_{a}\right)+c_{fix}\cdot T_{b}\right]$$ (1)

$$v_{a}=v_{a}^{free}{\left(1+0.15{\left(\frac{\sum_{b\in B}\sum_{r\in R}y_{s,r}^{b}\delta_{a,r}^{b}}{C_{a}}\right)}^{4}\right)}^{-1}$$ (2)

$$E_{b}\left(v_{a}\right)=k_{1}v_{a}^{2}+k_{2}$$ (3)

$$T_{b}=\sum_{r\in R}\sum_{s\in S}y_{s,r}^{b}\cdot \left(\frac{D_{r}}{v_{r}}+\tau_{charge}\cdot z_{b,t}\right)$$ (4)

Where $v_{a}$ denotes the link speed, $E_{b}\left(v_{a}\right)$ represents the energy consumption function, $E_{b}\left(v_{a}\right)$ denotes the time cost.

The second component encompasses the total travel costs for all affected passengers using various transportation modes, including temporal costs, economic costs, and comfort penalties:


$$\min F_{2}=\sum_{s\in S}{\tilde{d}}_{s}\cdot \sum_{m\in M}x_{s,m}^{p}\cdot \left[\alpha \cdot t_{s,m}+\beta \cdot c_{s,m}+\gamma \cdot g\left(w_{s,m}\right)\right]$$ (5)

The mode choice component employs a Random Regret Minimization (RRM) model to capture passengers’ behavioral responses when selecting:


$$x_{s,m}^{p}=\frac{\exp\left(-R_{s,m}/\sigma \right)}{\sum_{m^{\prime }\in M}\exp\left(-R_{s,m^{\prime }}/\sigma \right)}$$ (6)

$$R_{s,m}=\;\sum_{m^{\prime }\neq m}\ln\left(1+\exp\left(\mu \cdot \pi \left(V_{s,m^{\prime }}-V_{s,m}\right)\right)\right)$$ (7)

Where $V_{s,m}=\alpha t_{s,m}+\beta c_{s,m}$ is the deterministic utility term for mode m at station i, μ is the sensitivity parameter (regret aversion intensity).

Constraints:

(1) Demand Coverage Constraint: Ensures all passenger demand is assigned to at least one travel mode:


$$\sum_{m\in M}x_{s,m}^{p}=1,\forall s\in S$$ (8)

(2) Bus Capacity Constraint: The passenger load on buses cannot exceed the total vehicle supply capacity:


$$\sum_{s\in S}{\tilde{d}}_{s}x_{s,\text{bus}}^{p}\leq \sum_{b\in B}C_{b}\sum_{r\in R}y_{s,r}^{b}$$ (9)

(3) Charging Facility Constraint: The number of charging vehicles cannot exceed the maximum available charging points:


$$\sum_{b\in B}z_{b,t}\leq N_{charger},\forall t\in T$$ (10)

(4) Battery Dynamics Constraint: Tracks real-time battery level changes for electric buses:


$$Q_{b}^{t+1}=Q_{b}^{t}-\frac{L_{b}^{t}E_{b}\left(v_{a}\right)}{\eta }+\eta_{charge}z_{b,t}\Delta t$$ (11)

(5) Non-negative Frequency Constraint: Bus departure frequencies must be positive or zero:


$$y_{s,r}^{b}\geq 0,\forall s\in S,r\in R,b\in B$$ (12)

(6) Passenger Proportion Constraint: The proportion of passengers choosing each mode must be between 0 and 1:


$$0\leq x_{s,m}^{p}\leq 1,\forall s\in S,m\in M$$ (13)

## 3. Distributionally robust credibility optimization model

When dealing with the challenges of bus bridging services, especially in the face of unexpected event-induced ridership surges, the number of passengers stranded at a station due to differences in travel preferences (e.g., continuing with rail or switching to other modes of transportation) presents great uncertainty. Due to the lack of historical data, it is particularly difficult to accurately predict the passenger demand at each disruption station. For this kind of optimization problems with uncertainty, various solutions have been explored, among which stochastic and robust optimization methods are most commonly used [27]. Compared to stochastic optimization methods, robust optimization methods do not need to rely on specific possibility distributions of uncertain data, but rather work on finding solutions that remain optimal in the worst-case scenario Inspired by the study of Bertsimas and Sim [28], a parametric plausible optimization model is constructed by considering the uncertain passenger demand $d_{s}$ as an interval-valued fuzzy variable with a possibility distribution. Meanwhile, the decision maker usually requires the credibility of this constraint to satisfy a certain confidence level. The credibility measure Cr is essentially a trade-off operator between the possibility measure Pos and necessity measure Nec. This construction overcomes the excessive optimism of pure possibility constraints while avoiding the over-conservatism of necessity constraints. In demand model, the equivalent conversion between confidence level $\beta_{s}$ and credibility constraint is given by:


$$Cr\left\{d_{s}\geq {\tilde{d}}_{s}\right\}\geq \beta_{s},s\in S$$ (14)

The credibility constraint implies that, at least at the confidence level $\beta_{s}$, the number of passengers allowed on board at each station is greater than the passenger demand. According to the definition in the literature [29], let $\zeta_{s}$ be a parameter interval-valued fuzzy variable of ${\tilde{d}}_{s}$ with a second likelihood distribution of $\mu_{\zeta }\left(d_{s}\right)=\left[\mu_{\zeta^{L}}(d_{s};\theta_{l}),\mu_{\zeta^{K}}(d_{s};\theta_{k})\right]$, where $\mu_{\zeta^{L}}(d_{s};\theta_{l})$ is the lower choice variable and $\mu_{\zeta^{K}}(d_{s};\theta_{k})$ is the upper choice variable. Both $\theta_{l}$ and $\theta_{k}$ are fuzzy parameters, determined by the decision-maker based on expert experience or subjective judgment.

In order to characterize the fluctuation of the likelihood distribution $\mu_{\zeta }\left(d_{s}\right)$, a parameter selection variable $\zeta_{s,\lambda }$ is introduced to define the parameter interval value fuzzy variable chosen by λ, where $\lambda_{s}\in \left[0,1\right]$ reflects the decision maker’s optimistic or pessimistic decision-making attitude. Then the parametric likelihood distribution can be expressed as follows


$$\mu_{\zeta^{\lambda }}(d_{s};\theta )=(1-\lambda_{s})\mu_{\zeta^{L}}(d_{s};\theta_{l})+\lambda_{s}\mu_{\zeta^{K}}(d_{s};\theta_{k}),\theta =\left(\theta_{l},\theta_{k}\right)$$ (15)

where the values of $\theta_{l}$ and $\theta_{k}$ can be determined by the decision maker based on expert experience or subjective judgment.

According to the above description, the variation of parameter $\lambda_{s}$ determines the location of the likelihood distribution. That is, a plausibility constraint is a set of constraints with a common structure, i.e., with a fixed parameter $\lambda_{s}$, and with parameter $\lambda_{s}$ varying in a given set of uncertainty distributions $\mu_{\zeta }$ defined as follows:


$$\mu_{\zeta }=\left\{\mu_{\zeta^{\lambda }}(d_{s};\theta )|\mu_{\zeta^{\lambda }}(d_{s};\theta ),\lambda_{s}\in \left[0,1\right]\right\}$$ (16)

Based on this uncertainty set, the distributionally robust bus bridging service model can be expressed as:


$$\begin{array}{c} \left\{ \;\;\;\begin{array}{c} MinZ_{1}\\ MinZ_{2}\\ s.t.Cr\left(d_{s}\geq \zeta_{s}\right)\geq \beta_{s},\forall s\in S,\mu_{\zeta^{\lambda }}\left(d_{s};\theta \right) \end{array}\right. \end{array}$$ (17)

The credibility robust optimization model is transformed into a computationally solvable form. It is assumed that uncertain demands $\zeta_{s}$ are represented as parameter interval-valued trapezoidal fuzzy variables. Next, the transformation of the model is carried out in two steps: the analysis of the credibility constraints and the analysis of the robust peering.

### 3.1. Analysis of the credibility constraint

When passenger demand obeys a trapezoidal distribution, the analytical expression for the credibility constraint (12) can be expressed by the following theorem:

Theorem 1: Let passenger demand $\zeta_{s}=\left[r_{1}^{s},r_{2}^{s},r_{3}^{s},r_{4}^{s};\theta_{l}^{s},\theta_{k}^{s}\right]$ be expressed as a parameter interval-valued trapezoidal fuzzy variable. If $\zeta_{ij}$ are independent of each other, then the credibility constraint (12) can be transformed into the following form:

① When $\begin{array}{c} \beta_{s}\in \left(0,\frac{\lambda_{s}\theta_{k}^{s}-\left(1-\lambda_{s}\right)\theta_{l}^{s}+1}{\text{4}}\right] \end{array}$, then the plausibility constraint is equal to:


$$\frac{2\beta_{s}r_{2}^{s}+\left[\lambda_{s}\theta_{k}^{s}-\left(1-\lambda_{s}\right)\theta_{l}^{s}+1-2\beta_{s}\right]r_{1}^{s}}{1+\lambda_{s}\theta_{k}^{s}-\left(1-\lambda_{s}\right)\theta_{l}^{s}}\leq d_{s}$$ (18)

② When $\begin{array}{c} \beta_{s}\in \left(\frac{\lambda_{s}\theta_{k}^{s}-\left(1-\lambda_{s}\right)\theta_{l}^{s}+1}{\text{4}},\frac{\text{1}}{\text{2}}\right] \end{array}$, then the plausibility constraint is equal to:


$$\frac{\left[2\beta_{s}-\lambda_{s}\theta_{k}^{s}+\left(1-\lambda_{s}\right)\theta_{l}^{s}\right]r_{2}^{s}+\left(1-2\beta_{s}\right)r_{1}^{s}}{1-\lambda_{s}\theta_{k}^{s}+\left(1-\lambda_{s}\right)\theta_{l}^{s}}\leq d_{s}$$ (19)

③ When $\begin{array}{c} \beta_{s}\in \left(\frac{\text{1}}{\text{2}},\frac{3-\lambda_{s}\theta_{k}^{s}+\left(1-\lambda_{s}\right)\theta_{l}^{s}}{\text{4}}\right] \end{array}$, then the plausibility constraint is equal to:


$$\frac{\left(1-2\beta_{s}\right)r_{4}^{s}+\left[\lambda_{s}\theta_{k}^{s}-\left(1-\lambda_{s}\right)\theta_{l}^{s}-2+2\beta_{s}\right]r_{3}^{s}}{\lambda_{s}\theta_{k}^{s}-\left(1-\lambda_{s}\right)\theta_{l}^{s}-1}\leq d_{s}$$ (20)

④ When $\begin{array}{c} \beta_{s}\in \left(\frac{3-\lambda_{s}\theta_{k}^{s}+\left(1-\lambda_{s}\right)\theta_{l}^{s}}{\text{4}},1\right] \end{array}$, then the plausibility constraint is equal to:


$$\frac{\left[\lambda_{s}\theta_{k}^{s}-\left(1-\lambda_{s}\right)\theta_{l}^{s}-1+2\beta_{s}\right]r_{4}^{s}+(2-2\beta_{s})r_{3}^{s}}{1+\lambda_{s}\theta_{k}^{s}-\left(1-\lambda_{s}\right)\theta_{l}^{s}}\leq d_{s}$$ (21)

**Proof:** Since the uncertain demand $\zeta_{s}$ is a trapezoidal fuzzy variable of parameter interval values, $\zeta_{s}^{\lambda }$ it is a λ choice variable of $\zeta_{s}$. Therefore the parameter interval-valued likelihood distribution μ(d;θ,λ) of $\zeta_{s}$ is:


$$\begin{array}{c} \mu \left(d;\theta ,\lambda \right)\left\{ \begin{array}{c} @l\frac{\left[1+\lambda_{s}\theta_{k}^{s}-\left(1-\lambda_{s}\right)\theta_{i}^{s}\right]\left(\zeta_{s}^{\lambda }-r_{1}^{s}\right)}{r_{2}^{s}-r_{1}^{s}},\;\;r_{1}^{s}<\zeta_{s}^{\lambda }\leq \frac{r_{1}^{s}+r_{2}^{s}}{2}\\ \frac{\left[1-\lambda_{s}\theta_{r}^{s}+\left(1-\lambda_{s}\right)\theta_{l}^{s}\right]\zeta_{s}^{\lambda }+\left[\lambda_{s}\theta_{k}^{s}+\left(1-\lambda_{s}\right)\theta_{l}^{s}\right]r_{2}^{s}-r_{1}^{s}}{r_{2}^{s}-r_{1}^{s}},\frac{r_{1}^{s}+r_{2}^{s}}{2}<\zeta_{s}^{\lambda }\leq r_{2}^{s}\\ 1,\;r_{2}^{s}<\zeta_{s}^{\lambda }\leq r_{3}^{s}\\ \frac{\left[\lambda_{s}\theta_{r}^{s}-\left(1-\lambda_{s}\right)\theta_{l}^{s}-1\right]\zeta_{s}^{\lambda }-\left[\lambda_{s}\theta_{k}^{s}-\left(1-\lambda_{s}\right)\theta_{l}^{s}\right]r_{3}^{s}+r_{4}^{s}}{r_{4}^{s}-r_{3}^{s}},r_{3}^{s}<\zeta_{s}^{\lambda }\leq \frac{r_{3}^{s}+r_{4}^{s}}{2}\\ \frac{\left[1+\lambda_{s}\theta_{k}^{s}-\left(1-\lambda_{s}\right)\theta_{l}^{s}\right]\left(r_{4}^{s}-\zeta_{s}^{\lambda }\right)}{r_{4}^{s}-r_{3}^{s}},\;\;\frac{r_{3}^{s}+r_{4}^{s}}{2}<\zeta_{s}^{\lambda }\leq r_{4}^{s} \end{array}\right. \end{array}$$ (22)

According to the definition of plausibility measures [30], plausibility constraints can be handled in the following way:

When $\beta_{s}<0.5$, then:


$$Cr\left\{\zeta_{s}^{\lambda }\leq d_{s}\right\}=\frac{\text{1}}{\text{2}}\left\{1+\underset{d\leq u}{\sup}\mu \left(c^{\prime };\theta ,\lambda \right)-\underset{d>u}{\sup}\;\mu \left(c^{\prime };\theta ,\lambda \right)\right\}=\frac{\text{1}}{\text{2}}\underset{d\leq u}{\sup}\mu \left(c^{\prime };\theta ,\lambda \right)$$ (23)

Thus, the plausibility constraint $Cr\left\{d_{s}\geq \zeta_{s}^{\lambda }\right\}\geq \beta_{s}$ is equivalent to $\underset{d\leq u}{\sup}\mu \left(d;\theta ,\lambda \right)\geq 2\beta_{s}$, define $\zeta_{inf}\left(\theta \right)=\inf\left\{u|\underset{d\leq u}{\sup}\;\mu \left(d;\theta ,\lambda \right)\geq \beta_{s}\right\}$, where $\beta_{s}\in (0,1]$, then ${\bar{d}}_{inf}\left(2\beta_{s}\right)\leq u$.

If $\begin{array}{c} 0<2\beta_{s}\leq \frac{\left[1+\lambda_{s}\theta_{k}^{s}-\left(1-\lambda_{s}\right)\theta_{l}^{s}\right]}{\text{2}} \end{array}$, then $\begin{array}{c} \beta_{s}\in \left(0,\frac{\left[1+\lambda_{s}\theta_{k}^{s}-\left(1-\lambda_{s}\right)\theta_{l}^{s}\right]}{\text{4}}\right] \end{array}$, obtaining the (24):


$$\frac{\left[1+\lambda_{s}\theta_{k}^{s}-\left(1-\lambda_{s}\right)\theta_{l}^{s}\mathrm{\backslash rightleft}(\zeta_{s}^{\lambda }-r_{1}^{s}\right)}{r_{2}^{s}-r_{1}^{s}}=2\beta_{s}$$ (24)

Solving the Equation (24) gives:


$${\bar{d}}_{inf}\left(2\beta \right)=\frac{2\beta_{s}r_{2}^{s}+\left[\lambda_{s}\theta_{r}^{s}-\left(1-\lambda_{s}\right)\theta_{l}^{s}+1-2\beta_{s}\right]r_{1}^{s}}{1+\lambda_{s}\theta_{r}^{s}-\left(1-\lambda_{s}\right)\theta_{l}^{s}}$$ (25)

If $\begin{array}{c} \frac{\left[1+\lambda_{s}\theta_{r}^{s}-\left(1-\lambda_{s}\right)\theta_{l}^{s}\right]}{\text{2}}<2\beta_{s}\leq 1 \end{array}$, then $\begin{array}{c} \beta_{s}\in \left(\frac{\left[1+\lambda_{s}\theta_{r}^{s}-\left(1-\lambda_{s}\right)\theta_{l}^{s}\right]}{\text{4}},\frac{\text{1}}{\text{2}}\right] \end{array}$, obtaining the Equation (26):


$$\frac{\left[1-\lambda_{s}\theta_{r}^{s}+\left(1-\lambda_{s}\right)\theta_{l}^{s}\right]c_{s}+\left[\lambda_{s}\theta_{r}^{s}+\left(1-\lambda_{s}\right)\theta_{l}^{s}\right]r_{2}^{s}-r_{1}^{s}}{r_{2}^{s}-r_{1}^{s}}=2\beta_{s}$$ (26)

Solving the Equation (26) yields Equation (27)


$${\bar{d}}_{inf}\left(2\beta \right)=\frac{\left[2\beta_{s}-\lambda_{s}\theta_{r}^{s}+\left(1-\lambda_{s}\right)\theta_{l}^{s}\right]r_{2}^{s}+\left(1-2\beta_{s}\right)r_{1}^{s}}{1-\lambda_{s}\theta_{r}^{s}+\left(1-\lambda_{s}\right)\theta_{l}^{s}}$$ (27)

The proofs of ① and ② are completed, and ③ and ④ in Theorem 1 can be proved in a similar way.

To simplify the expression, denote the segmented functions in Theorem 1 by $\pi_{\beta }\left(d;\theta ,\lambda \right)$ and denote $D_{\beta }^{n}$ as the domain of definition of each segmented function, then the plausibility constraint can be expressed as:


$$\pi_{\beta }\left(d;\theta ,\lambda \right)\leq d_{s},\forall s\in S,\beta_{s}\in D_{\beta }^{n}$$ (28)

### 3.2. Analysis of robust equivalence

Since the given set of uncertainty distributions $U_{\zeta }$ is a continuous set, Equation (29) actually represents a semi-infinite programming model containing infinitely many constraints. In order to solve such complex models, they need to be transformed into a computationally tractable form, and the core idea of robust optimization, which is to find a solution that remains feasible for all possible realizations of the uncertain parameters, can be rewritten for Equation (29):


$$\underset{\mu_{\zeta^{\lambda }}(d_{s};\theta )\in U_{\zeta }}{\max}\left\{\pi_{\beta }\left(d;\theta ,\lambda \right)\right\}\leq d_{s},\forall s\in S,\beta_{s}\in D_{\beta }^{n}$$ (29)

To solve the above problem, the maximization operation at the left end of the inequality in the original problem is transformed into a dyadic problem by means of a dyadic transformation [31]. The original inequality (24) is equivalently expressed in the form of a segmented function:


$$\pi_{\beta }^{*}\left(d;\theta \right)=\left\{ \begin{array}{c} \frac{2\beta_{s}r_{2}^{s}+\left(1-\theta_{l}^{s}-2\beta_{s}\right)r_{1}^{s}}{1-\theta_{l}^{s}}\leq q_{s},\beta_{s}\in (0,\frac{1-\theta_{l}^{ij}}{\text{4}}\backslash \backslash frac\left(2\beta_{s}+\theta_{l}^{s}\right)r_{2}^{s}+\left(1-2\beta_{l}^{s}\right)r_{1}^{s}1+\theta_{l}^{s}\leq q_{s},\beta_{s}\in \left(\frac{1-\theta_{l}^{s}}{\text{4}},\frac{\text{1}}{\text{2}}\right]\\ \frac{\left(1-2\beta_{s}\right)r_{4}^{s}+\left(\theta_{r}^{s}-2+2\beta_{s}\right)r_{3}^{s}}{\theta_{r}^{s}-1}\leq q_{s},\beta_{s}\in \left(\frac{\text{1}}{\text{2}},\frac{3-\theta_{r}^{s}}{\text{4}}\right]\\ \frac{\left(\theta_{r}^{s}-1+2\beta_{s}\right)r_{4}^{s}+\left(2-2\beta_{s}\right)r_{3}^{s}}{1+\theta_{r}^{s}}\leq q_{s},\beta_{s}\in \left(\frac{3-\theta_{r}^{s}}{\text{4}},1\right] \end{array}\right.$$ (30)

The first two inequalities are obtained from λ=0, while the last two inequalities are obtained from λ=1. Based on the above analysis, an equivalent deterministic model can be obtained:


$$\left\{ \begin{array}{c} MinF_{1}\\ MinF_{2}\\ s.t.\pi_{\beta }^{*}\left(d;\theta \right)\leq d_{s},\forall s\in S,\beta_{s}\in D_{\beta }^{n} \end{array}\right.$$ (31)

## 4. Reinforcement learning-based parallel genetic algorithm

Multi-objective optimization problems are highly challenging due to their NP-hard nature, making it difficult for exact algorithms to find feasible solutions within reasonable timeframes. To address this challenge, researchers have developed a series of heuristic algorithms—such as iterative greedy algorithms, evolutionary algorithms, and particle swarm optimization—which demonstrate excellent performance when dealing with problems featuring relatively simple constraints. However, their effectiveness often becomes limited when confronted with highly complex problems. To break through this bottleneck, many scholars have leveraged advanced technologies like parallel computing to further refine these algorithms.

In pursuit of high-quality solutions, this study designs a Reinforcement-learning-based Parallel Genetic Algorithm (RPGA), which achieves innovative breakthroughs in three key aspects compared to existing methods:

(1) Dynamic Parameter Adaptation via Q-Learning

Traditional genetic algorithms typically rely on fixed rules or empirical formulas to adjust parameters (e.g., linearly decreasing crossover and mutation probabilities), which struggle to adapt to the dynamic changes in complex solution spaces inherent to multi-objective optimization. RPGA innovatively incorporates Q-Learning to dynamically adjust crossover probability, mutation probability, and the methods for crossover and mutation operations [15].

(2) Heterogeneous Population Architecture

Conventional genetic algorithms employ a single homogeneous population, which is prone to premature convergence to local optima. RPGA adopts a master-slave island model, designing a parallel computing framework that accelerates the evolutionary process through genetic operations and population communication strategies [19].

The fundamental differences between RPGA and NSGA-II are summarized in Table 3:

**Table 3:** Comparison between NSGA-II and RPGA.

Comparison item	NSGA-II	RPGA
Parameter Control Mode	Relies on manually preset fixed parameters and cannot adapt to changing problem characteristics	Utilizes Q-learning to autonomously select crossover/mutation operations, forming self-adaptive evolutionary strategies
Population Structure and Search Logic	Employs a homogeneous population with random crossover/mutation, exhibiting limitations in blind search	Implements master-slave heterogeneous population collaboration: the master island employs reinforcement learning for global strategy decisions while slave islands perform dedicated exploration/exploitation tasks, achieving optimal balance between directed search and diversity maintenance
Computational Paradigm and Efficiency	Serial iteration results in prolonged computation time, particularly inefficient for high-dimensional problems	Implements multi-island asynchronous evolution via parallel architecture combined with elite migration mechanism, significantly reducing computational time
Constraint Handling Capability	Uses static penalty functions requiring repeated weight coefficient adjustments	Automatically guides population evolution toward feasible regions through constraint violation terms in the reward function, eliminating manual parameter tuning

**Fig 3:**
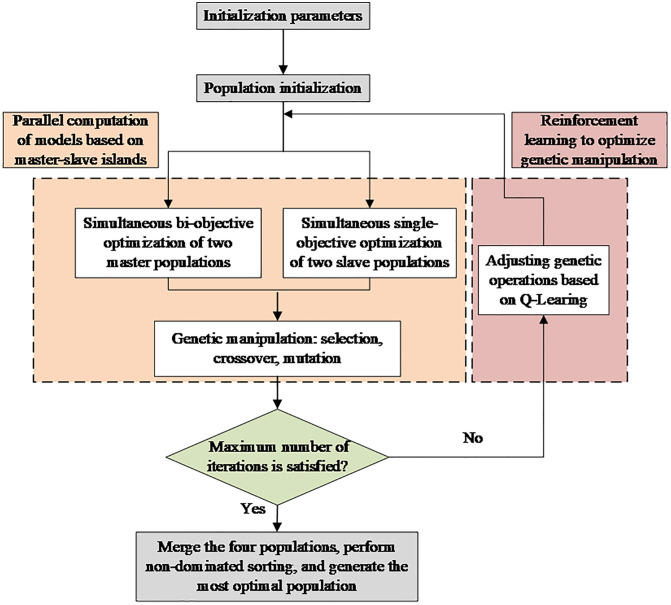
Flowchart of PRGA algorithm.

Fig 3 presents the detailed flowchart of the RPGA algorithm, which starts with population initialization and progresses through multiple stages including genetic operations, population communication, and reinforcement learning-based parameter adaptation, ultimately outputting high-quality solution sets.

### 4.1. Parallel computing based on master-slave island model

**Fig 4:**
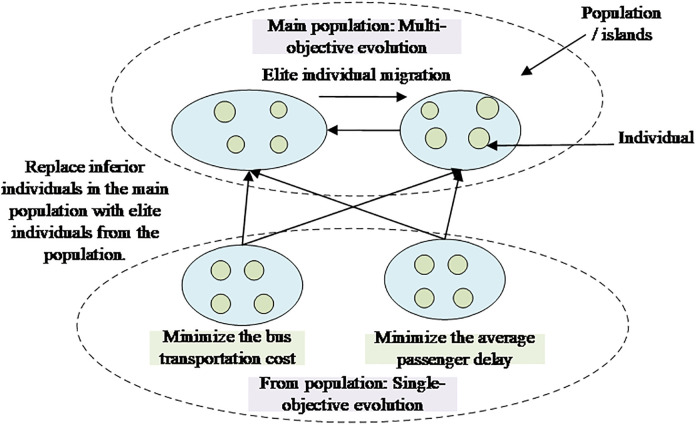
Structural diagram of PPCS.

The master-slave island model represents an efficient parallel computing architecture that divides the population into master and slave populations for parallel evolution. During the evolutionary process, the master population engages in regular exchanges of elite individuals, while the slave population contributes high-quality individuals to the master population. This setup fosters knowledge sharing and evolutionary dynamics among the populations. Given the complexity of the constraints in the BBSDP (a complex and multi-objective optimization problem), it is challenging for a single population to effectively approximate the true Pareto frontier. To address this, we propose the Population Parallel Computing Strategy (PPCS) based on the master-slave island model, as illustrated in Fig 4. This strategy establishes two master populations for multi-objective search and two slave populations for single-objective search, focusing on the two optimization objectives of BBSD. All populations simultaneously perform genetic operations, including selection, crossover, and mutation, to drive the evolutionary process. During iteration, bidirectional communication is maintained between the master populations, while the slave populations communicate unidirectionally to the master populations. This arrangement enhances the probability of the master populations approaching the true Pareto frontier.

### 4.2. Reinforcement learning to optimize genetic manipulation

**Fig 5:**
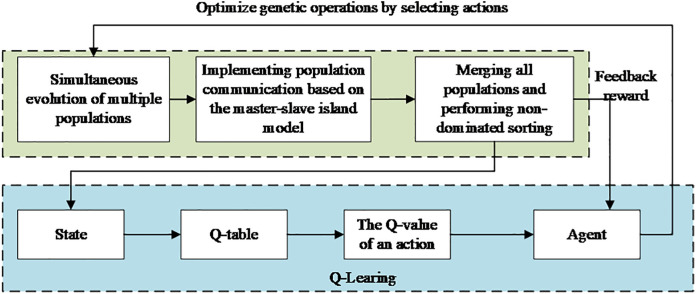
The process of optimizing RPGA by Q-Learning.

The fixed genetic operation strategy in genetic algorithms often results in the algorithm becoming trapped in local optimal solutions, thereby limiting its global search capability. To address this issue, this study introduces the Q-Learning algorithm from the reinforcement learning mechanism to dynamically adjust the parameters of genetic operations, thereby enhancing the algorithm’s global optimization performance. The state and reward mechanisms of Q-Learning are intimately tied to the evolutionary quality of the population. Utilizing the Q-Table, the algorithm selects the optimal action (i.e., the adjustment strategy for the genetic operation parameters) based on the current state. After each iteration, the evolution of the population is fed back to the Q-Learning algorithm to update the Q values in the Q-Table. Fig 5 illustrates the specific flow of the Q-Learning optimized genetic operation, where the design of the state set, action set, and reward process is crucial for the implementation of the algorithm.

Based on the algorithmic strategy introduced earlier, the pseudocode is designed as follows

**Algorithm 1 Reinforcement-guided Parallel Genetic Algorithm (RPGA)**

Input:Population size $N_{p}$; Maximum generations $T_{\max}$; Migration rate RM; Migration interval PM; Number of islands $N_{i}$; Crossover probability $P_{c}$; Mutation probability $P_{m}$; Genetic operators O

Output:Approximated Pareto front $P_{front}$

Steps:

1 **Initialization**

2  P ←RandomPopulation($N_{P}$)

3  Partition P into $N_{i}$ subpopulations $P_{i},i\in N_{i}$

4  Initialize RL agent with state space S and action space A

5  T ←1, converged←False

6 **end Initialization**

7 **while** $T<T_{\max}$ **and not** converged **do**

8  **Parallel Evolution on Islands**:

9   **for each** subpopulation $P_{i}$ **in parallel do**

10   Perform selection: $P_{i}$ ←Selection$\left(P_{i},O\right)$ ▷ *Tournament selection*

11   Perform crossover: $P_{i}$ ←Crossover$\left(P_{i},P_{c}\right)$ ▷ *SBX crossover*

12   Perform mutation: $P_{i}$ ←Mutation$\left(P_{i},P_{m}\right)$ ▷ *Polynomial mutation*

13   Evaluate fitness: EvaluateFitness$\left(P_{i}\right)$

14  **end for**

15 **Migration & RL Adaptation**:

16  **if** t mod PM ==0 **then**

17   ▷ *Ring-topology migration*

18   **for** i=1 **to** $N_{i}$ **do**

19    Migrate $RM\cdot N_{i}$ individuals from $P_{i}$ to $P_{\left(iodN_{i}\right)+1}$

20   **end for**

21  **end if**

22  ▷ *Reinforcement learning adaptation*

23  **for each** $P_{i}$ **do**

24   Compute state: $s_{t}$ ←ComputeState $\left(P_{i}^{t},P_{i}^{t-1}\right)$ ▷ *State: Population metrics*

25   **if** ConvergenceCheck ($s_{t}$,threshold=5%) **then**

26    Set converged←True

27   **else**

28    Compute reward: $r_{t}$ ←RewardFunction $\left(s_{t}\right)$ ▷ *Based on HV/IGD improvement*

29    Update policy: $a_{t}$ ←RLPolicyUpdate $\left(s_{t},r_{t}\right)$ ▷ *Adjust* $P_{c},P_{m},O$

30    Update $P_{c},P_{m},O$ with action $a_{t}$

31   **end if**

32  **end for**

33  Increment generation: t ←t+1

34 **end while**

35 **return** NonDominated Sort$\left(\cup_{i=1}^{N_{i}}P_{i}\right)$ ▷ *Final Pareto front*

## 5. Numerical experiments

### 5.1. Network description

**Fig 6:**
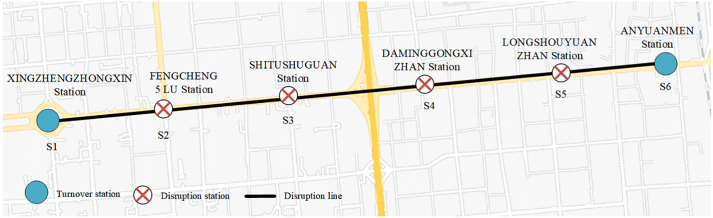
Xi’an metro line 2 disruption stations.

Considering the operational disruption on Xi’an Metro Line 2 during the 08:00–10:00, which lasted for 2 hours and affected 6 stations (including two terminal stations, S1 and S6 in S1 File, and four disrupted stations, S2, S3, S4, and S5 in S1 File), we need to devise an efficient bus bridging service. The bus bridging service, with a rated passenger capacity of 50 people per bus and a departure interval of 5 minutes, aims to replace the disrupted section of the metro line and facilitate passenger travel between the affected stations (S2, S3, S4, and S5 in S1 File). There are five dispatch points in the vicinity ready to provide the necessary transportation. The topology of the metro line is depicted in Fig 6, while Table 4 outlines the number of vehicles available for dispatch from each point and the distances to the respective emergency bus stations.

**Table 4:** Number of vehicles that can be dispatched and the distance to each bus station.

Dispatch point	Number of buses (vehicles)	Distance (km)					
		S1	S2	S3	S4	S5	S6
1	10	3.5	4.8	4.5	4.8	5.2	6.1
2	10	5.2	4.7	3.9	5.4	6.5	3.3
3	10	4.6	5.2	5.8	6.2	4.0	4.8
4	10	5.3	4.2	3.8	5.0	6.3	3.9

This study utilizes the Automatic Fare Collection (AFC) system swipe card data from Xi’an Metro Line 2 during May 1–7, 2021, employing systematic data cleaning and preprocessing methods. First, quality control is performed on the raw data to remove incomplete records with missing key fields (e.g., exit station and timestamp information). Second, temporal logic anomalies (such as exit times preceding entry times and other chronological errors) are corrected. Concurrently, abnormal trips are identified and excluded based on trip duration characteristics (outliers with dwell times <1 minute or >4 hours). On this basis, an origin-destination (OD) passenger flow matrix with a 15-minute temporal resolution is constructed to effectively extract spatiotemporal distribution characteristics of historical passenger flows, thereby providing high-quality data foundation for subsequent modeling research.

According to the construction method of fuzzy affiliation function, the trapezoidal parameter $\left(r_{1}^{s},r_{2}^{s},r_{3}^{s},r_{4}^{s}\right)$ estimated based on the historical data of passenger flow is obtained, as shown in Table 5. Uncertain demand is denoted by $\left(r_{1}^{s},r_{2}^{s},r_{3}^{s},r_{4}^{s},\theta_{l}^{s},\theta_{k}^{s}\right)$, where $\theta_{l}^{s}$ and $\theta_{k}^{s}$ are determined by the decision maker based on expert experience or subjective judgment. In this study, the values of parameters $\theta_{l}$ and $\theta_{k}$ are 0.24 and 0.15, respectively. Meanwhile, the confidence level is set to be $\beta_{s}=0.9$. To guarantee a high degree of confidence in the evacuation process, we compare the numerical results obtained from the nominal model with those derived from the proposed distributionally robust credibility model. In this comparison, the nominal demand for each origin-destination (OD) pair is set equal to the average value of the trapezoidal parameters.

**Table 5:** Running times and corresponding trapezoidal parameter values for OD pairs.

OD	Running time (minutes)	Trapezoidal parameters			
(1,2)	3	196	218	237	259
(1,3)	7	87	98	104	112
(1,4)	12	58	63	73	84
(1,5)	16	49	54	60	67
(1,6)	20	65	79	88	94
(2,3)	4	171	217	226	243
(2,4)	8	144	233	254	270
(2,5)	13	206	228	254	279
(2,6)	17	203	223	242	278
(3,4)	3	93	107	118	127
(3,5)	6	78	88	99	107
(3,6)	10	118	129	142	156
(4,5)	4	51	57	63	69
(4,6)	8	74	83	92	102
(5,6)	12	62	69	77	84

### 5.2. Parameter settings

The values of relevant parameters in the model are shown in the Table 6:

**Table 6:** Parameter value.

Parameter	Value	Parameter	Value
Electricity cost ψ	1.2 RMB/kWh	Energy coefficient $k_{1}$	0.003 kWh/(km(km/h)^2^)
Bus fixed cost $c_{fix}$	200 RMB/h	Energy constant $k_{2}$	0.8 kWh/km
Taxi fare rate $C_{taxi}$	12 + 3 RMB/km	Battery capacity $Q_{b}^{\max}$	300 kWh
Bike-sharing fee $C_{bike}$	2 RMB/h	Charging efficiency $\eta_{c}$	90%
Time value coefficient α	20 RMB/h	Motor efficiency $\eta_{m}$	90%
Cost sensitivity factor β	0.15	Bus passenger capacity $C_{b}$	50 person/trip
Regret sensitivity μ	1.5	BPR coefficients α 、β	0.15, 4
Comfort penalty weight γ	0.1	Number of chargers	5 unit
Link capacity $C_{a}$	1500 veh/h		

In order to determine the optimal values of the key parameters of RPGA, experiments are conducted on the main population evolution process based on reinforcement learning to determine the optimal parameters. 600 samples are randomly selected for each experiment, and the evaluation index is taken as the mean value of the results of 10 experiments [32]. The optimal parameter configuration for the Q-Learning algorithm is determined as follows: the discount factor is set to 0.85 through grid search (test range [0.7,0.99]); an exponential decay strategy from 1.0 to 0.1 is adopted for the exploration rate; and an adaptive adjustment mechanism from 0.6 to 0.1 is implemented for the learning rate, significantly improving training stability. The optimal parameter values of the algorithm are finally derived as follows: RM=0.3, PM=50, $N_{p}=120$, $T_{\max}=200$, $N_{i}=4$, $P_{c}\in \left[0.80,0.95\right]$,$P_{m}\in \left[0.02,0.2\right]$.

### 5.3. Results and discussion

The comparative results between the nominal model and distributionally robust credibility model are presented in Tables 7 and 8. The nominal model dispatched 28 buses to handle station disruptions, generating 58,765 RMB in vehicle transportation costs and 27,580 RMB in passenger travel costs. In comparison, the distributionally robust model only required 23 buses, reducing emergency vehicle costs by 16.1% to 49,326 RMB while incurring a modest 3.1% increase in passenger costs to 28,430 RMB. This represents a reduction of 5 vehicles deployed and significant savings of 9,439 RMB in transportation costs, offset by a slight 850 RMB rise in passenger expenses. By explicitly accounting for demand uncertainty through marginally increased transfer times, the distributionally robust model demonstrates superior robustness in shuttle solutions under uncertain conditions. The findings validate its enhanced adaptability and cost-effectiveness in developing emergency bus scheduling strategies when facing demand fluctuations, making it particularly suitable for practical transit disruption management applications.

**Table 7:** Nominal model solution results.

Comparison term	Dispatch point				Total
	D1	D2	D3	D4	
The number of dispatched buses (vehicle)	8	9	5	6	28
Buses transportation costs (RMB)	16790	18889	10494	12593	58765
Passenger travel costs (RMB)	7446.6	8549.8	5516	6067.6	27580

**Table 8:** Distributionally robust credibility model solution results.

Comparison term	Dispatch point				Total
	D1	D2	D3	D4	
The number of dispatched buses (vehicle)	6	7	5	5	23
Buses transportation costs (RMB)	12868	15012	10723	10723	49326
Passenger travel costs (RMB)	7391.8	9079.6	6254.6	5686	28430

#### 5.3.1. Comparative analysis of algorithms.

1) **Comparative Analysis of RPGA Algorithm versus exact optimization solver CPLEX**

**Fig 7:**
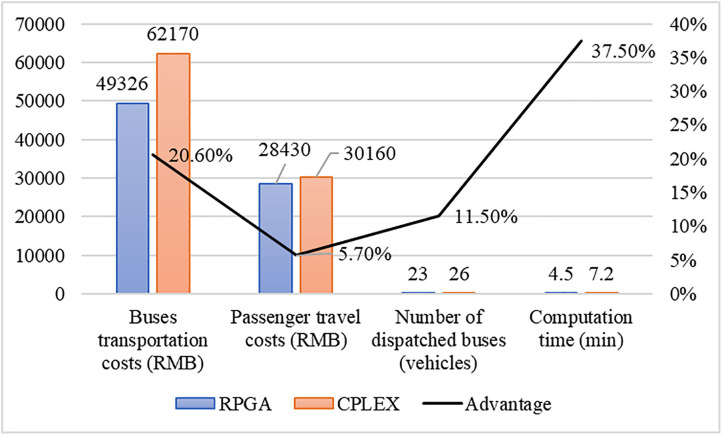
Comparative analysis between RPGA and the exact solver CPLEX.

This study systematically compares the performance of the RPGA algorithm with the exact solver CPLEX (results shown in Fig 7), yielding the following key findings: In the bus bridging optimization problem, the RPGA algorithm demonstrates significant comprehensive advantages: (1) Economically, RPGA achieves a 20.6% reduction in buses transportation costs and a 5.7% decrease in passenger travel costs; (2) In terms of resource allocation, the required number of dispatched buses is reduced by 11.5%; (3) Regarding computational efficiency, the computation time is shortened by 37.5% compared to CPLEX. These improvements are primarily attributed to the heuristic strategy adopted by RPGA, which effectively circumvents the combinatorial explosion problem. When addressing large-scale real-time scheduling problems, RPGA significantly enhances computational speed while ensuring solution quality.

2) **Comparison between RPGA Algorithm and Multi-objective Optimization Algorithms**

This study compares RPGA with three multi-objective optimization algorithms: NSGA-II [33], Reinforcement Learning-based NSGA-II (RLNSGA-II) [34], and Multi-objective Particle Swarm Optimization Algorithm (MOPSO) [35]. Notably, RPGA’s encoding scheme was also implemented in NSGA-II and MOPSO for fair comparison. To comprehensively evaluate algorithm performance, four established metrics were employed: Hypervolume (HV) measuring solution diversity and convergence, Inverted Generational Distance (IGD) assessing convergence quality, Non-Dominated Ratio (NDR) quantifying solution dominance, and computational TIME recording algorithm efficiency.

① The HV metric provides a comprehensive evaluation of algorithm performance by simultaneously considering three key aspects: cardinality, accuracy, and diversity. A larger HV value indicates superior population quality. The metric is formally calculated as follows:


$$HV=\delta \left(\cup_{i=1}^{\left|S\right|}v_{i}\right)$$ (32)

Where δ represents the Lebesgue measure, S denotes the non-dominated solution set. |S| is the size of the non-dominated set. $v_{i}$ denotes the hypervolume formed between the reference point and the i -th solution in the solution set.

② The IGD serves as a superior metric for evaluating population convergence, where a smaller IGD value indicates better population quality. The metric is computed as follows:


IGD=(∑r∈R{mindq∈Q(q,r)})/(∑r∈R{mindq∈Q(q,r)})|R|\nulldelimiterspace|R| (33)

Where R denotes the reference set. |R| represents the cardinality of the reference set. d(q,r) is the minimum Euclidean distance between a reference point r and all solutions q in the non-dominated solution set Q.

③ The NDR is employed to compare the quality of non-dominated solutions across different algorithms. A higher NDR value indicates superior algorithm performance. Using RPGA as an example, the calculation proceeds through the following steps:

Step 1: Merge the non-dominated solution sets obtained from running all four algorithms, denoted as S.

Step 2: Perform non-dominated sorting on S to identify the global non-dominated set ND. Let $ND_{PRGA}$ denote the subset of ND originating from RPGA.

Step 3: Let || denote the cardinality (size) of a set, NDRPRGA=|NDPRGA|/|NDPRGA||ND|\nulldelimiterspace|ND|.

For the benchmark algorithms, certain parameters were set identical to RPGA while others followed values from the literature. Specifically in NSGA-II, the crossover and mutation probabilities matched RPGA’s settings with other parameters set according to published studies. Similarly for RLNSGA-II, five key parameters including learning rates and operator probabilities were aligned with RPGA while maintaining literature-based values for remaining parameters [28]. MOPSO’s parameters were configured strictly reference [29] implementations.

**Fig 8:**
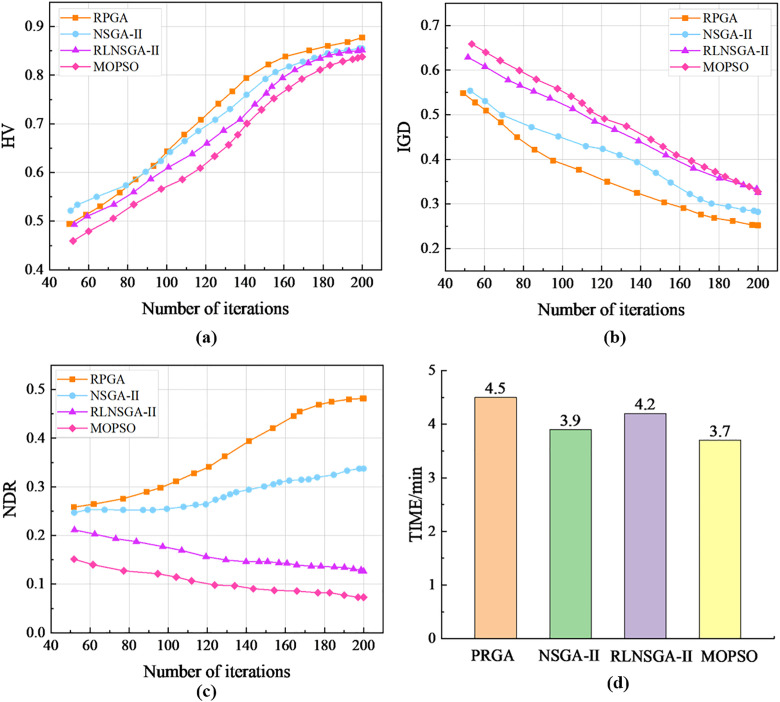
Trend of algorithm performance metrics: (a)HV; (b)IGD; (c)NDR; (d)TIME.

The evaluation protocol consisted of 20 independent experimental runs for each algorithm. Performance metrics including HV, IGD and NDR were averaged across runs and visualized through trend line plots, with computational time (TIME/min) recorded for each method. As shown in the results Fig 8, our analysis leads to the following key conclusions:

(1) Overall, RPGA demonstrates superior performance in HV, IGD, and NDR metrics compared to other algorithms while maintaining TIME within an acceptable range. In the early iterations, RPGA’s metrics are comparable to NSGA-II and RLNSGA-II but significantly outperform MOPSO, indicating that genetic evolution-based algorithms are more suitable for MAVRP. As iterations progress, RPGA’s metrics optimize rapidly and show clear advantages over other algorithms, with RLNSGA-II ranking second, demonstrating that reinforcement learning can significantly enhance algorithmic search capability. The consistent superiority of RPGA over RLNSGA-II proves the effectiveness of the master-slave island model’s parallel computing in further improving the quality of non-dominated solutions.
(2) After approximately 110 iterations, RPGA’s HV, IGD, and NDR metrics become markedly superior to other algorithms. By 160 iterations, RPGA’s metric curves begin to stabilize, whereas other algorithms only start stabilizing after 180 iterations – and even then at inferior metric levels compared to RPGA. This clearly indicates RPGA’s higher search efficiency.
(3) The computation time (TIME) of RPGA is comparable to RLNSGA-II but slightly longer than NSGA-II and MOPSO, primarily due to the additional computational overhead introduced by reinforcement learning. However, considering the overall performance, RPGA demonstrates significantly superior results in HV, IGD, and NDR metrics compared to other algorithms. Essentially, RPGA achieves higher-quality solutions at the cost of marginally increased computation time.

To comparatively analyze the performance of RPGA against NSGA-II, RLNSGA-II, and MOPSO in solving multi-objective scheduling models, this study conducted a series of experiments. As shown in Table 9. Regarding buses transportation costs, RPGA achieved superior results at 49,326 RMB, representing a 16.1% reduction compared to the second-best performer NSGA-II (58,765 RMB). For passenger travel costs, RPGA attained 28,430 RMB, a 3.9% improvement over NSGA-II (29,580 RMB), demonstrating simultaneous optimization of both cost dimensions. Furthermore, RPGA required only 23 buses for scheduling – 4.2% fewer than MOPSO (24 buses) and the lowest among all algorithms, confirming its capability for resource-efficient scheduling.

**Table 9:** Comparison of optimization results of related indicators.

Comparison Item	RPGA	NSGA-II	RLNSGA-II	MOPSO
Buses transportation costs (RMB)	49326	58765	60350	59820
Passenger travel costs (RMB)	28430	29580	29700	30460
Number of dispatched buses (vehicles)	23	25	26	24

#### 5.3.2. Impact of confidence level on optimal shuttle decision-making.

The above results were obtained under a confidence level $\beta_{s}=0.9$. To further investigate the impact of $\beta_{s}$ on the optimal shuttle decision, we fixed parameters $\theta_{l}=0.25$ and $\theta_{k}=0.16$ and varied $\beta_{s}$ within the interval [0.5,0.9] with a step size of 0.1. To evaluate the utility of the distributionally robust credibility model under different uncertain demand scenarios, we considered a surge in passenger flow at disrupted stations by selecting nominal demand deviation values of 0.20, 0.25, 0.30, 0.35, and 0.40 to represent varying degrees of demand fluctuations. The computational results are presented in Table 10, where the expected unmet rate and expected occupancy rate are weighted averages calculated under both nominal and maximum deviation demand values.

From Table 10, it can be observed that for any fixed deviation value, the expected unmet rate of the distributionally robust credibility model increases as the confidence level $\beta_{s}$ decreases, while the expected occupancy rate decreases. This implies that higher confidence levels require additional costs from bus operators, such as providing more shuttle vehicles or increasing service frequency, to meet a larger proportion of passenger demand. Conversely, for any fixed confidence level $\beta_{s}$, both the expected unmet rate and expected occupancy rate increase with higher deviation values. This indicates that greater demand variability leads to higher vehicle occupancy, but when vehicles reach capacity, the number of unmet passenger demands also rises. To mitigate potential risks, the maximum confidence level $\beta_{s}$ across different paths was used to calculate the expected unmet rate and expected occupancy rate, ensuring robustness under extreme demand fluctuations.

**Table 10:** Expected occupancy rate and expected unmet rate under different confidence levels and deviation values.

Deviation Value		Expected occupancy rate					Expected unmet rate				
		0.20	0.25	0.30	0.35	0.40	0.20	0.25	0.30	0.35	0.40
$\beta_{s}$	0.9	0.864	0.865	0.865	0.868	0.868	0.014	0.017	0.019	0.024	0.027
	0.8	0.921	0.922	0.923	0.924	0.924	0.015	0.021	0.026	0.033	0.039
	0.7	0.963	0.965	0.966	0.967	0.968	0.026	0.036	0.046	0.055	0.064
	0.6	0.975	0.976	0.976	0.977	0.978	0.044	0.058	0.071	0.083	0.089
	0.5	0.986	0.988	0.988	0.989	0.99	0.053	0.067	0.076	0.091	0.095

**Fig 9:**
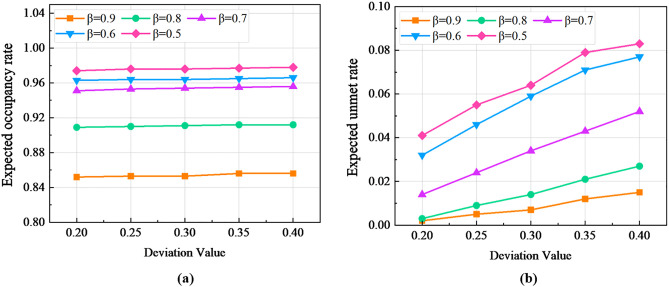
The trends of expected occupancy rate and expected unmet rate under different demand deviation values across five confidence levels: (a) Expected occupancy rate; (b) Expected unmet rate.

Fig. 9 illustrates the trends of expected occupancy rate and expected unmet rate under different demand deviation values across five confidence levels. As shown in Fig 9a, the growth rate of the expected occupancy rate decreases as the confidence level increases, indicating that shuttle vehicles tend to reach full capacity as demand rises. This suggests that higher confidence levels require more resources to accommodate increasing passenger demand, but the marginal gain in occupancy diminishes. In contrast, Fig 9b reveals that the growth rate of the expected unmet rate increases with higher confidence levels. This implies that lower confidence levels result in a higher proportion of unmet demand due to insufficient capacity, highlighting the instability of solutions under such conditions.

#### 5.3.3. Rated passenger capacity analysis.

**Fig 10:**
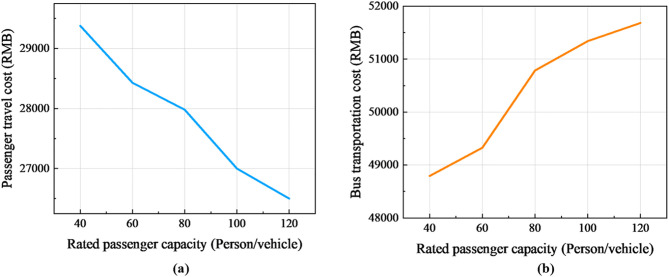
Rated passenger capacity analysis: (a) Rated passenger capacity-bus transportation costs; (b) Rated passenger capacity-average passenger delay.

The relationship between the rated passenger capacity and bus transportation costs and average passenger delays is explored, as shown in Fig 10. In a certain interval, as the rated passenger capacity increases, the bus transportation cost tends to increase, while the average passenger delay decreases accordingly. This indicates that the adjustment of rated passenger capacity has a significant effect on the implementation of the rescue program.

## 6. Conclusion

This paper investigates the BBS in response to URT disruption/'.pk;. s and introduces a bus bridging model that incorporates the uncertainty of passenger demand. A multi-objective emergency bus scheduling model is developed, aiming to minimize both bus transportation costs and average passenger delays. Given the significant uncertainty in the number of passengers stranded at stations, the uncertain demand is modeled as a trapezoidal fuzzy variable, leading to the establishment of a distributionally robust credibility optimization model. To solve this model, a parallel genetic algorithm enhanced by reinforcement learning is proposed. The study is validated using a case study of the Xi’an Metro Line 1 section between Cao Tan Station and Chang Ning Gong Station during the morning peak hours of 07:00–09:00. The key findings are as follows:

(1) Comparative analysis between the nominal model and the distributionally robust credibility model reveals that the latter achieves superior operational efficiency: it reduces the number of dispatched vehicles by 3 (12.5% decrease), lowers emergency transportation costs by 9,439 RMB (16.1% reduction), while accepting a marginal 3.1% increase (850 RMB) in passenger costs. This trade-off stems from the model’s explicit consideration of demand uncertainty, which yields more robust shuttle solutions through strategically extended transfer times.
(2) Comprehensive evaluation demonstrates RPGA’s dominance across all performance metrics: it achieves 4% higher hypervolume (HV), 20% improved inverted generational distance (IGD), and 2.5 × greater non-dominated ratio (NDR) compared to NSGA-II, RLNSGA-II and MOPSO algorithms, with only a 10–15% computational time overhead. These advantages translate to concrete operational benefits: 49,326 RMB in transportation costs (16.1% lower than NSGA-II’s 58,765 RMB) and 28,430 RMB in passenger costs (3.9% reduction versus NSGA-II’s 29,580 RMB), conclusively validating its superiority in multi-objective optimization.
(3) The trend of expected full load rate and expected unmet rate under different demand deviation values at five confidence levels is analyzed. With the increase of confidence level, the growth rate of expected full load rate decreases, indicating that the feeder vehicles tend to be full-load with the increase of demand. With the increase of the confidence level, the growth rate of the expected unmet rate increases, indicating that when the co nfidence level is low, the larger passenger demand will lead to a higher proportion of unserved demand, and the stability is poor.
(4) Analysis of the rated passenger capacity of emergency vehicles reveals that within a certain range, an increase in rated capacity leads to a corresponding rise in bus transportation costs and a decrease in passenger travel costs, demonstrating that rated passenger capacity significantly impacts rescue plans.

The proposed model has several areas that warrant further improve ment. Firstly, this study focuses solely on disruptions occurring in a single URT route. Future research could expand this scope by developing a model that addresses disruptions across an entire URT network. Secondly, the current approach designs the bridging service in a static environment, which overlooks the dynamic nature of passenger flow propagation and public transport interactions within the spatiotemporal network. To address this limitation, future work could explore the design of bus bridging services within a spatiotemporal network framework, enabling a more accurate representation of real-world conditions.

## Supporting information

S1 File May 1st −7st Metro Line 2 OD Statistics Table. (XLSX)
